# Sleep duration and age-related macular degeneration: a cross-sectional and Mendelian randomization study

**DOI:** 10.3389/fnagi.2023.1247413

**Published:** 2023-08-22

**Authors:** Shizhen Lei, Zhouyang Liu, Haihui Li

**Affiliations:** ^1^Department of Ophthalmology, The First Hospital of Wuhan, Tongji Medical College, Huazhong University of Science and Technology, Wuhan, Hubei, China; ^2^Department of Neurology, The Third People’s Hospital of Chengdu, Chengdu, Sichuan, China; ^3^Department of Ophthalmology, Yan’an People’s Hospital, Yan’an, Shaanxi, China

**Keywords:** sleep, age-related macular degeneration, cross-sectional study, Mendelian randomization, causal effect

## Abstract

**Purpose:**

To investigate the association between sleep duration and age-related macular degeneration (AMD).

**Design:**

Cross-sectional study, bidirectional two-sample Mendelian randomization (MR). For cross-sectional analysis, we used survey data of 5,481 participants aged ≥40 years from the 2005 to 2008 National Health and Nutrition Examination Survey (NHANES). For MR analysis, we used sleep- and AMD-associated genome-wide association studies (GWAS) data involving large populations.

**Methods:**

The association between sleep duration and AMD was assessed using logistic regression models. For MR analysis, the primary approach for MR analysis was the inverse-variance weighted (IVW) method.

**Results:**

In cross-sectional analysis, after adjusting for multiple covariates, short sleep duration (SSD) was found to be associated with increased risk of early AMD [odds ratio (OR) = 1.364, *P* = 0.036). MR analysis supported the results of cross-sectional analysis: SSD increases the risk of early AMD (β = 0.102, IVW-*P* = 0.003).

**Conclusion:**

Our findings provide the evidence supporting the association between sleep deficiency and higher risk of AMD. Further studies are required to confirm our findings and elucidate the mechanisms underlying this association.

## 1. Introduction

Age-related macular degeneration (AMD) is a neurodegenerative disease (NDD) involving neuroretina and retinal pigment epithelium (RPE), thereby leading to visual impairment or even blindness (Wong et al., 2014). Studies have reported considerable health burden in patients with AMD, which mainly affects adults aged 40 years and older (Taylor et al., 2016; Zhu et al., 2019). The number of patients with AMD is continuously increasing and estimated to be about 288 million worldwide by 2040 (Congdon et al., 2004; Wong et al., 2014). Previous studies have suggested that oxidative stress, inflammation and RPE senescence may all play a critical role (Donoso et al., 2010; Kauppinen et al., 2016), however, the pathogenesis of AMD is still not fully understood.

Sleep disorders have been considered as a hallmark of aging and elders have increasing difficulty in falling and staying asleep with advancing age (Ohayon, 2002). Particularly, sleep problems are frequently observed in patients with NDDs including Alzheimer’s disease (AD) (Lin et al., 2013), and those with cardiovascular diseases such as hypertension and coronary artery disease (Javaheri and Redline, 2017; Pandi-Perumal et al., 2017). Notably, AMD shares some common risk factors with these NDDs and cardiovascular diseases.

The National Health and Nutrition Examination Survey (NHANES) is a nationally conducted survey of the non-institutionalized civilian population in the United States. Previous studies have explored the risk or protective factors of AMD based on the NHANES data (Obisesan et al., 1998; Zhu et al., 2020). In this study, we explored the association between sleep duration (SD) and AMD using the survey data from NHANES.

Observational studies are vulnerable to reverse causation, residual confounding, and selective bias (Grimes and Schulz, 2002), which may negatively influence the estimates of relationship between SD and AMD. A randomized clinical trial (RCT) allows reliable and robust causal inferences to be drawn, but it is costly, time-consuming, and sometimes impractical to conduct one. Mendelian randomization (MR) approaches have opened up opportunities to assess and determine clinically associated characters for multiple diseases (Davey Smith and Hemani, 2014), which examines causal relationships between exposures and outcomes using genetic variants significantly associated with an exposure as instrumental variables. By using MR approaches, these biases can be nicely overcome (Davey Smith and Hemani, 2014).

Though there is increasing evidence indicating the correlation between sleep disorder and AMD, the underlying mechanisms are not identified yet and the evidence from observational studies is very limited. In this study, we aimed to comprehensively investigate the potential role of sleep abnormalities in the pathogenesis of AMD. As a result, cross-sectional and MR analysis indicated that sleep deprivation increased the risk of AMD. In conclusion, the results indicated that sleep deficiency could increase the risk of AMD.

## 2. Materials and methods

### 2.1. Cross-sectional study design

#### 2.1.1. Participants

**FIGURE 1:**
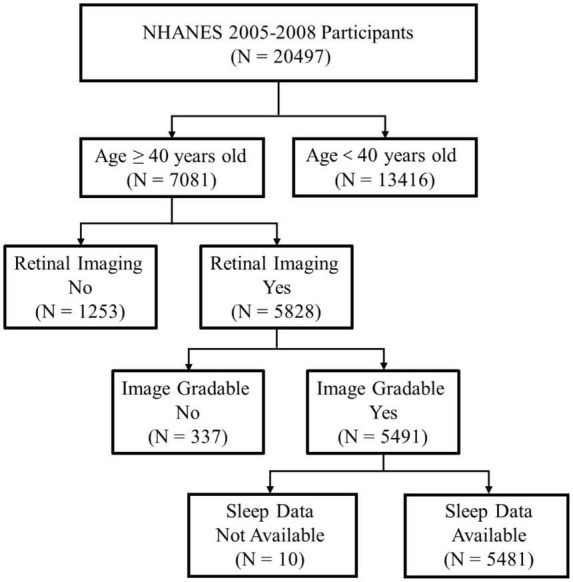
Schematic diagram showing study participants included for the present analysis from the 2005 to 2008 NHANES. A total of 5,481 participants aged 40 years and older with valid fundus photographs and available information on sleep status were included. NHANES, National Health and Nutrition Examination Survey.

National Health and Nutrition Examination Survey data collected from two study cycles (2005–2006 and 2007–2008) was used in this study. Firstly, we identified total 7,081 participants aged 40 years and older. Subsequently, we excluded 1,253 participants without necessary retinal photographs and 337 participants without information on classification of AMD severity. Furthermore, 10 participants without sleep data were excluded. After that, the final 5,481 participants were identified (Figure 1). In the NHANES surveys, participants were asked to provide written informed consent before being enrolled.

#### 2.1.2. Assessment of retinal photography and AMD severity

Retinal photographs of the final sample (5,481 participants) were collected. The assessment of the retinal photographs has been completed and provided on the NHANES website (Klein et al., 2011). The diagnosis and classification of AMD was conducted by at least two experienced experts following a strict procedure. The definition of early AMD: signs of drusen with a grid area of >500 μm circle and/or pigmentary abnormalities. Late/advanced AMD was defined as the presence of exudative or geographic atrophy signs. If retinal photographs of both eyes were available, the status of the eye with higher AMD severity would be used in analyses.

#### 2.1.3. Definition of sleep status

Abnormal SD was defined in the following way:

Insufficient/short sleep duration (SSD) (<7 h per night), normal (7–8 h per night), or excessive/long sleep duration (LSD) (≥9 h per night) (Plantinga et al., 2012; Sleep Foundation, 2016).

#### 2.1.4. Covariates

The regressions models were adjusted for covariates that have been associated with SD and AMD: age, gender, race/ethnicity, education level, marital status, income level, alcohol consumption, smoking status, body mass index (BMI), self-reported history of hypertension, hyperlipidemia, diabetes mellitus, osteoporosis, general health condition, glaucoma, cataract surgery, and cardiovascular disease (CVD). Race were divided as non-Hispanic White and the others. Education level was categorized as those who had completed less than high school, completed high school or graduate equivalency degree, and completed more than high school. Income level is defined based on the poverty income ratio (PIR), with values <1.5 representing lower income, ≥1.5 and <3 representing middle income and ≥3 representing higher income. Alcohol consumption was categorized as no alcohol use, moderate (women: 1 drink per day or men: 1–2 drinks per day), heavy (women: 2–3 drinks per day or men: 3–4 drinks per day) and binge (women: ≥4 drinks per day or men: ≥5 drinks per day), according to definitions from the National Institute on Alcohol Abuse and Alcoholism. Smoking status was defined as low (blood cotinine <0.015 ng/ml), high (blood cotinine 0.015–3 ng/ml), and high (blood cotinine >3 ng/ml). BMI is calculated by the weight divided by height squared (kg/m^2^) and defined as not overweight (BMI <25 kg/m^2^), overweight (BMI 25–30 kg/m^2^) and obesity (BMI ≥30 kg/m^2^). Self-reported history of CVD was defined as having a previous physician diagnosis of congestive heart failure, coronary heart disease, angina, heart attack, or stroke.

### 2.2. Bidirectional MR study design

#### 2.2.1. Data source for exposures and outcomes

In our study, we implemented bidirectional two-sample MR to judge causation using summary statistics from independent and large genome-wide association studies (GWAS) on SSD and LSD (Dashti et al., 2019) and AMD (Fritsche et al., 2016; Winkler et al., 2020). Summary-level statistics for early AMD from Winkler et al. (2020) contains 105,248 participants in European population (14,034 early AMD cases; 91,214 controls). The International Age-related Macular Degeneration Genomics Consortium (IAMDGC) contains 16,144 advanced AMD patients and 17,832 controls of European ancestry (Fritsche et al., 2016). An ideal two-sample MR analysis requires a condition where no overlap between exposure and outcome participants occurs. Participants of exposures and outcomes are hardly overlapped and details regarding the characteristics of individual studies included in the consortium have been described in their website and the published paper (Fritsche et al., 2016; Dashti et al., 2019; Winkler et al., 2020).

#### 2.2.2. Selection of genetic instruments

**FIGURE 2:**
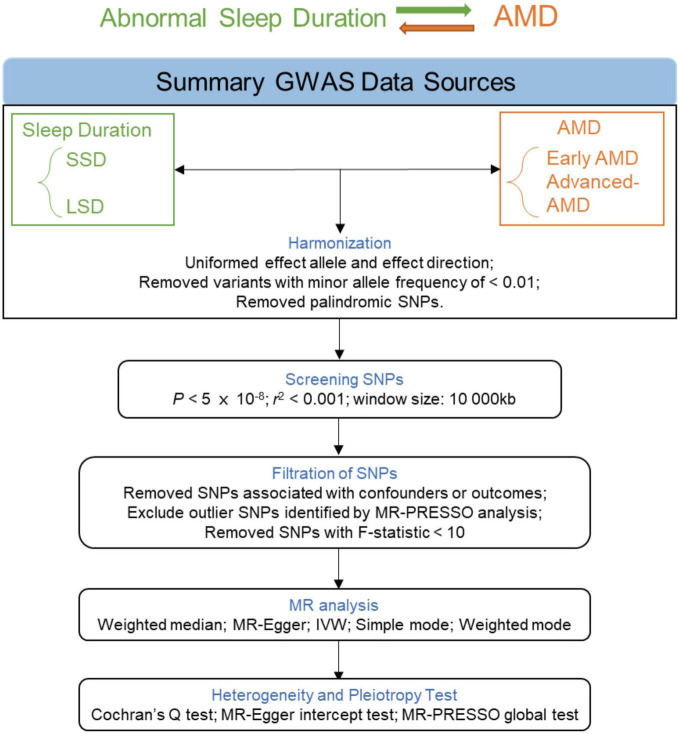
Flowchart of the MR analyses. MR, Mendelian randomization; GWAS, genome-wide association study; LSD, long sleep duration; SSD, short sleep duration; AMD, age-related macular degeneration; SNP, single nucleotide polymorphism; PRESSO, Pleiotropy RESidual Sum and Outlier; IVW, inverse-variance weighted.

Instrumental variables (single nucleotide polymorphisms, SNPs) were selected via the following criteria: (i) with genome-wide significance (*P* < 5 × 10^–8^) and (ii) pruned by linkage disequilibrium (*r*^2^ < 0.001 and within 10,000 kb from the index variant). MR Pleiotropy RESidual Sum and Outlier (MR-PRESSO) (Verbanck et al., 2018) was used to remove the underlying outlier SNPs, thereby enhancing the robustness of the results. The *F* statistics of these SNPs were also taken into consideration. PhenoScanner (Staley et al., 2016) is an online platform with comprehensive information about genotype-phenotype association. We examined whether the obtained instrumental SNPs were associated with the outcomes and the potential confounders and subsequently remove the associated ones. The flowchart of MR analysis is presented in Figure 2.

#### 2.2.3. MR approaches

Mendelian randomization’s validity depends on the crucial assumption of no pleiotropy (Lawlor et al., 2008). Therefore, we used five MR approaches [random-effect inverse-variance weighted (IVW), MR Egger, weighted median, simple mode, and weighted mode] to address the heterogeneity and pleiotropy effect in estimating. The IVW method (Burgess et al., 2016) was taken as the primary one, whereas the other methods were used to improve the robustness of IVW-derived results as what they are designed for Bowden et al. (2016), Hartwig et al. (2017). We performed Cochran’s *Q* test to evaluate the heterogeneity and detect pleiotropy (Bowden et al., 2019). Besides, the MR-PRESSO (Verbanck et al., 2018) test was used to further assess the robustness of the causal effect between SD and AMD and SNPs identified as outliers by MR-PRESSO were removed. We also conducted leave-one-out analyses and MR-Egger intercept test to assess horizontal pleiotropy for significant estimates. In addition, we utilized a funnel plot to detect possible directional pleiotropy, similarly to how publication bias is assessed in a meta-analysis.

### 2.3. Statistical analyses

Statistical analyses were performed using R software (version 4.0.1) and EmpowerStats software. Continuous variables were expressed as means ± standard deviations (SD). Categorical variables were summarized as the counts or percentages (%).

For analysis of clinical data, EmpowerStats software and logistic regression model was used to calculate adjusted odd ratios (OR) and 95% confidence intervals (CIs). Three models were evaluated for outcomes: model 1 was unadjusted; model 2 was adjusted for age and gender; model 3 was further adjusted for the rest of covariates. Adjusted *P* < 0.05 was taken as significant.

For MR analysis, we performed all the analyses in R (version 4.0.1) using the TwoSampleMR (Hemani et al., 2018) and MR-PRESSO (Verbanck et al., 2018) R packages. All statistical tests are two-sided. *P* < 0.05 was considered as nominally significant and *P* < 0.05/6 (8.33 × 10^–3^) (Bonferroni-corrected *P*) was considered as significant.

## 3. Results

### 3.1. Cross-sectional analysis

The characteristics of the study population are presented in Tables 1, 2. Among the participants, 77.2% were white people and 52.8% were females. Approximately 55.8, 65.1, and 35.5% of the people reported that they had completed more than high school, got married, and overweight, respectively. Almost 39.8% of participants reported an SSD status, whereas only 6.8% of participants reported an LSD status.

**TABLE 1:** Demographic characteristics, health-related behaviors, and comorbidities of participants with and without abnormal sleep duration (SD).

Characteristics	Overall (*N* = 5,481)	Short SD (*N* = 2,181)	Normal SD (*N* = 2,929)	Long SD (*N* = 371)	*P*-value
Age (%)					<0.001
40–64	75.2 (73.9–76.5)	79.3 (77.4–81.1)	74.9 (73.2–76.6)	53.2 (47–59.3)	
65–79	19.7 (18.6–20.9)	16.5 (14.9–18.3)	20.2 (18.7–21.8)	34.6 (29.1–40.6)	
≥80	5.1 (4.6–5.6)	4.2 (3.5–5)	4.9 (4.3–5.6)	12.2 (9.3–15.8)	
Gender (%)					<0.001
Male	47.2 (45.5–48.9)	49.4 (46.7–52.1)	47.1 (44.8–49.4)	34.8 (29.2–40.8)	
Female	52.8 (51.1–54.5)	50.6 (47.9–53.3)	52.9 (50.6–55.2)	65.2 (59.2–70.8)	
Race (%)					<0.001
Non-Hispanic White	77.2 (76–78.3)	69.6 (67.4–71.7)	81.6 (80.1–83)	81.9 (77.8–85.3)	
Others	22.8 (21.7–24)	30.4 (28.3–32.6)	18.4 (17–19.9)	18.1 (14.7–22.2)	
Education (%)					<0.001
Less than high school	17.9 (16.8–19)	19.6 (17.9–21.5)	15.9 (14.5–17.4)	25.3 (20.6–30.6)	
High school or graduate equivalency degree	26.3 (24.9–27.9)	28.1 (25.7–30.6)	25.2 (23.3–27.2)	26 (20.8–32.1)	
More than high school	55.8 (54.1–57.4)	52.3 (49.6–54.9)	58.9 (56.7–61.1)	48.7 (42.4–55)	
Marital status (%)					<0.001
Unmarried	34.9 (33.4–36.5)	40.3 (37.7–42.9)	31.1 (29.1–33.1)	38.3 (32.5–44.3)	
Married	65.1 (63.5–66.6)	59.7 (57.1–62.3)	68.9 (66.9–70.9)	61.7 (55.7–67.5)	
Income level (%)					<0.001
Lower income	18.7 (17.6–19.8)	22.2 (20.3–24.2)	15.9 (14.6–17.4)	22.9 (18.5–28)	
Middle income	24.1 (22.7–25.6)	24.1 (21.8–26.4)	22.9 (21–24.8)	36.2 (30.2–42.6)	
Higher income	57.2 (55.5–58.9)	53.7 (51–56.5)	61.2 (59–63.4)	40.9 (34.5–47.7)	
Smoking status (%)					<0.001
Low	20.3 (19–21.8)	18.6 (16.5–20.9)	21.7 (19.8–23.7)	18.3 (13.6–24.1)	
Moderate	54.2 (52.5–55.9)	51.9 (49.2–54.7)	55.5 (53.2–57.8)	55.1 (48.6–61.4)	
High	25.5 (24–27)	29.5 (27–32)	22.8 (20.9–24.8)	26.6 (21.3–32.8)	
Alcohol use (%)					0.032
Moderate	59.2 (57.1–61.3)	56.5 (53–59.9)	60.7 (57.9–63.4)	61 (52.3–69)	
Heavy	30.1 (28.2–32.2)	31.5 (28.3–34.9)	29 (26.5–31.7)	32.7 (25.1–41.3)	
Binge	10.7 (9.4–12)	12 (9.9–14.4)	10.3 (8.7–12)	6.3 (3.2–12.3)	
BMI (%)					<0.001
<25 kg/m^2^, not overweight	27.7 (26.2–29.2)	23.8 (21.6–26.2)	29.7 (27.6–31.8)	32.3 (26.6–38.5)	
25–30 kg/m^2^, overweight	35.5 (33.9–37.2)	36.5 (33.9–39.1)	35.1 (33–37.3)	33.4 (27.7–39.5)	
≥30 kg/m^2^, obesity	36.8 (35.2–38.4)	39.7 (37.1–42.4)	35.2 (33–37.4)	34.3 (28.6–40.5)	
Hypertension (%)					<0.001
No	59 (57.3–60.6)	57.1 (54.5–59.7)	60.9 (58.7–63.1)	52 (45.7–58.2)	
Yes	41 (39.4–42.7)	42.9 (40.3–45.5)	39.1 (36.9–41.3)	48 (41.8–54.3)	
Hyperlipidemia (%)					0.059
No	51.3 (49.4–53.1)	49.9 (46.9–52.8)	52.7 (50.2–55.1)	46.9 (40.2–53.8)	
Yes	48.7 (46.9–50.6)	50.1 (47.2–53.1)	47.3 (44.9–49.8)	53.1 (46.2–59.8)	
Diabetes (%)					<0.001
No	89.5 (88.5–90.4)	87.3 (85.6–88.9)	91 (89.8–92.1)	88.3 (84.4–91.2)	
Yes	10.5 (9.6–11.5)	12.7 (11.1–14.4)	9 (7.9–10.2)	11.7 (8.8–15.6)	
Cataract surgery (%)					<0.001
No	91 (90.2–91.8)	92.1 (90.8–93.2)	91.4 (90.3–92.4)	80.9 (76–85)	
Yes	9 (8.2–9.8)	7.9 (6.8–9.2)	8.6 (7.6–9.7)	19.1 (15–24)	
Glaucoma (%)					<0.001
No	95.7 (95.1–96.3)	95.5 (94.3–96.4)	96.5 (95.7–97.1)	89.9 (85.6–93.1)	
Yes	4.3 (3.7–4.9)	4.5 (3.6–5.7)	3.5 (2.9–4.3)	10.1 (6.9–14.4)	
Health condition (%)					<0.001
Good or very good	81.6 (80.3–82.7)	76.6 (74.4–78.6)	85.5 (83.9–86.9)	75.8 (70.2–80.7)	
Fair	15.3 (14.2–16.4)	19.4 (17.5–21.5)	12.1 (10.9–13.6)	19.2 (14.8–24.5)	
Poor	3.7 (2.7–3.7)	4 (3.2–5)	2.4 (1.8–3.1)	5 (3–8.3)	
Osteoporosis (%)					0.166
No	92.4 (91.5–93.2)	92 (90.5–93.2)	92.9 (91.7–93.9)	90.3 (86.4–93.3)	
Yes	7.6 (6.8–8.5)	8 (6.8–9.5)	7.1 (6.1–8.3)	9.7 (6.7–13.6)	
CVD (%)					0.052
No	82.1 (80.8–83.3)	81.8 (79.8–83.7)	82.8 (81–84.3)	77.5 (72.4–81.9)	
Yes	17.9 (16.7–19.2)	18.2 (16.3–20.2)	17.2 (15.7–19)	22.5 (18.1–27.6)	

BMI, body mass index; CVD, cardiovascular disease.

**TABLE 2:** Demographic characteristics, health-related behaviors, and comorbidities of participants with and without age-related macular degeneration (AMD).

Characteristics	Overall (*N* = 5,481)	No AMD (*N* = 5,052)	Early AMD (*N* = 374)	Late AMD (*N* = 55)	*P*-value
Age (%)					<0.001
40–64	75.2 (73.9–76.5)	78 (76.7–79.2)	39.3 (32.9–46.1)	7.4 (2.8–18.1)	
65–79	19.7 (18.6–20.9)	18.5 (17.3–19.7)	39 (33.2–45.1)	28.1 (16.6–43.4)	
≥80	5.1 (4.6–5.6)	3.5 (3.1–4)	21.7 (17.7–26.4)	64.5 (49.5–77.1)	
Gender (%)					0.055
Male	47.2 (45.5–48.9)	47.2 (45.5–49)	49.4 (43.1–55.8)	30.1 (19.1–44.1)	
Female	52.8 (51.1–54.5)	52.8 (51–54.5)	50.6 (44.2–56.9)	69.9 (55.9–80.9)	
Race (%)					<0.001
Non-Hispanic White	77.2 (76–78.3)	76.5 (75.3–77.7)	85.5 (81.7–88.6)	94.4 (87–97.7)	
Others	22.8 (21.7–24)	23.5 (22.3–24.7)	14.5 (11.4–18.3)	5.6 (2.3–13)	
Education (%)					0.195
Less than high school	17.9 (16.8–19)	17.6 (16.4–18.7)	22.4 (17.9–27.6)	20.8 (12.1–33.5)	
High school or graduate equivalency degree	26.3 (24.9–27.9)	26.3 (24.8–27.9)	26 (20.9–31.8)	31.3 (19.6–46.1)	
More than high school	55.8 (54.1–57.4)	56.1 (54.4–57.8)	51.6 (45.3–57.9)	47.9 (34.1–62)	
Marital status (%)					<0.001
Unmarried	34.9 (33.4–36.5)	34.4 (32.8–36.1)	38.8 (33.1–44.9)	65.5 (50.9–77.6)	
Married	65.1 (63.5–66.6)	65.6 (63.9–67.2)	61.2 (55.1–66.9)	34.5 (22.4–49.1)	
Income level (%)					<0.001
Lower income	18.7 (17.6–19.8)	18.2 (17.1–19.4)	23.9 (19.2–29.4)	35.9 (23.7–50.2)	
Middle income	24.1 (22.7–25.6)	23.7 (22.2–25.2)	30.9 (25.3–37.1)	32 (19.7–47.4)	
Higher income	57.2 (55.5–58.9)	58.1 (56.4–59.9)	45.2 (38.5–52)	32.1 (19.6–47.9)	
Smoking status (%)					0.026
Low	20.3 (19–21.8)	20 (18.6–21.5)	25.9 (20.7–31.9)	17.6 (9.2–31.1)	
Moderate	54.2 (52.5–55.9)	54.3 (52.5–56.1)	50.3 (43.9–56.7)	69.7 (55.3–81.1)	
High	25.5 (24–27)	25.7 (24.2–27.3)	23.8 (18.4–30.1)	12.7 (5.9–25.2)	
Alcohol use (%)					0.005
Moderate	59.2 (57.1–61.3)	58.5 (56.3–60.7)	70.5 (62.2–77.6)	68.5 (43.9–85.8)	
Heavy	30.1 (28.2–32.2)	30.6 (28.5–32.7)	24.2 (17.6–32.3)	13.2 (3.2–41.5)	
Binge	10.7 (9.4–12)	10.9 (9.6–12.3)	5.3 (2.7–10.2)	18.3 (6.6–41.5)	
BMI (%)					0.029
<25 kg/m^2^, not overweight	27.7 (26.2–29.2)	27.5 (25.9–29.1)	28.7 (23.1–35)	39 (26–53.8)	
25–30 kg/m^2^, overweight	35.5 (33.9–37.2)	35.3 (33.6–37)	38.3 (32.4–44.6)	45.1 (31.5–59.5)	
≥30 kg/m^2^, obesity	36.8 (35.2–38.4)	37.2 (35.5–38.9)	33 (27.3–39.2)	15.9 (8.4–27.9)	
Hypertension (%)					<0.001
No	59 (57.3–60.6)	59.7 (58–61.4)	49.6 (43.3–55.9)	40.3 (27.3–54.7)	
Yes	41 (39.4–42.7)	40.3 (38.6–42)	50.4 (44.1–56.7)	59.7 (45.3–72.7)	
Hyperlipidemia (%)					0.721
No	51.3 (49.4–53.1)	51.4 (49.5–53.3)	50.4 (43.6–57.1)	45.3 (30.4–61)	
Yes	48.7 (46.9–50.6)	48.6 (46.7–50.5)	49.6 (42.9–56.4)	54.7 (39–69.6)	
Diabetes (%)					0.336
No	89.5 (88.5–90.4)	89.6 (88.6–90.5)	87.8 (83.4–91.2)	84.5 (70.1–92.7)	
Yes	10.5 (9.6–11.5)	10.4 (9.5–11.4)	12.2 (8.8–16.6)	15.5 (7.3–29.9)	
Cataract surgery (%)					<0.001
No	91 (90.2–91.8)	92.4 (91.6–93.1)	76.4 (71.3–80.8)	35.6 (23.3–50.1)	
Yes	9 (8.2–9.8)	7.6 (6.9–8.4)	23.6 (19.2–28.7)	64.4 (49.9–76.7)	
Glaucoma (%)					<0.001
No	95.7 (95.1–96.3)	96.1 (95.5–96.7)	90.2 (85.9–93.3)	84.5 (71.2–92.3)	
Yes	4.3 (3.7–4.9)	3.9 (3.3–4.5)	9.8 (6.7–14.1)	15.5 (7.7–28.8)	
Health condition (%)					0.135
Good or very good	81.6 (80.3–82.7)	81.8 (80.5—83)	76.9 (71.6–81.5)	86.9 (73.8–94)	
Fair	15.3 (14.2–16.4)	15 (13.9–16.2)	19.9 (15.6–25)	13.1 (6–26.2)	
Poor	3.7 (2.7–3.7)	3.2 (2.7–3.8)	3.2 (1.9–5.4)	–	
Osteoporosis (%)					<0.001
No	92.4 (91.5–93.2)	92.8 (91.9–93.6)	87.3 (82.8–90.7)	79.5 (65.1–89)	
Yes	7.6 (6.8–8.5)	7.2 (6.4–8.1)	12.7 (9.3–17.2)	20.5 (11–34.9)	
CVD (%)					<0.001
No	82.1 (80.8–83.3)	83.1 (81.8–84.3)	70.2 (64.4–75.5)	52 (37.9–65.7)	
Yes	17.9 (16.7–19.2)	16.9 (15.7–18.2)	29.8 (24.5–35.6)	48 (34.3–62.1)	

BMI, body mass index; CVD, cardiovascular disease.

Three logistic regression models were used to explore the relationship between SD and AMD. When taking AMD as the outcome, a significantly positive association were observed between SSD and AMD (OR = 1.364, 95% CI: 1.058–1.941) in the multivariable logistic model (Table 3). When abnormal SD was considered as the outcome, there was a significant association between AMD and SSD in model 1 (OR = 1.944, 95% CI: 1.418–2.666); however, after adjusting for multiple covariates (model 3), no significant association between AMD and SSD was observed (Table 4).

**TABLE 3:** Logistic regression models of abnormal sleep duration for age-related macular degeneration (AMD).

Variables	Model 1^a^ OR (95% CI)	*P*-value	Model 2^b^ OR (95% CI)	*P*-value	Model 3^c^ OR (95% CI)	*P*-value
Short sleep duration	1.977 (1.34–2.917)	<0.001	1.388 (1.179–1.969)	0.007	1.364 (1.058–1.941)	0.036
Long sleep duration	1.011 (0.777–1.316)	0.934	1.17 (0.936–1.463)	0.169	1.13 (0.901–1.417)	0.29

^a^Model 1: unadjusted. ^b^Model 2: adjusted for age and gender. ^c^Model 3: further adjusted for race/ethnicity, education level, marital status, income level, alcohol consumption, smoking status, body mass index (BMI), self-reported history of hypertension, hyperlipidemia, diabetes mellitus, osteoporosis, general health condition, glaucoma, cataract surgery, and cardiovascular disease (CVD).

**TABLE 4:** Logistic regression models of age-related macular degeneration (AMD) for abnormal sleep duration (SD).

Outcomes	Model 1^a^ OR (95% CI)	*P*-value	Model 2^b^ OR (95% CI)	*P*-value	Model 3^c^ OR (95% CI)	*P*-value
Short sleep duration	1.944 (1.418–2.666)	<0.001	1.343 (0.958–1.883)	0.087	1.334 (0.947–1.878)	0.099
Long sleep duration	0.738 (0.572–0.951)	0.019	0.93 (0.702–1.232)	0.614	0.963 (0.733–1.267)	0.79

^a^Model 1: unadjusted. ^b^Model 2: adjusted for age and gender. ^c^Model 3: further adjusted for race/ethnicity, education level, marital status, income level, alcohol consumption, smoking status, body mass index (BMI), self-reported history of hypertension, hyperlipidemia, diabetes mellitus, osteoporosis, general health condition, glaucoma, cataract surgery, and cardiovascular disease (CVD).

### 3.2. MR analysis of SD and AMD

**FIGURE 3:**
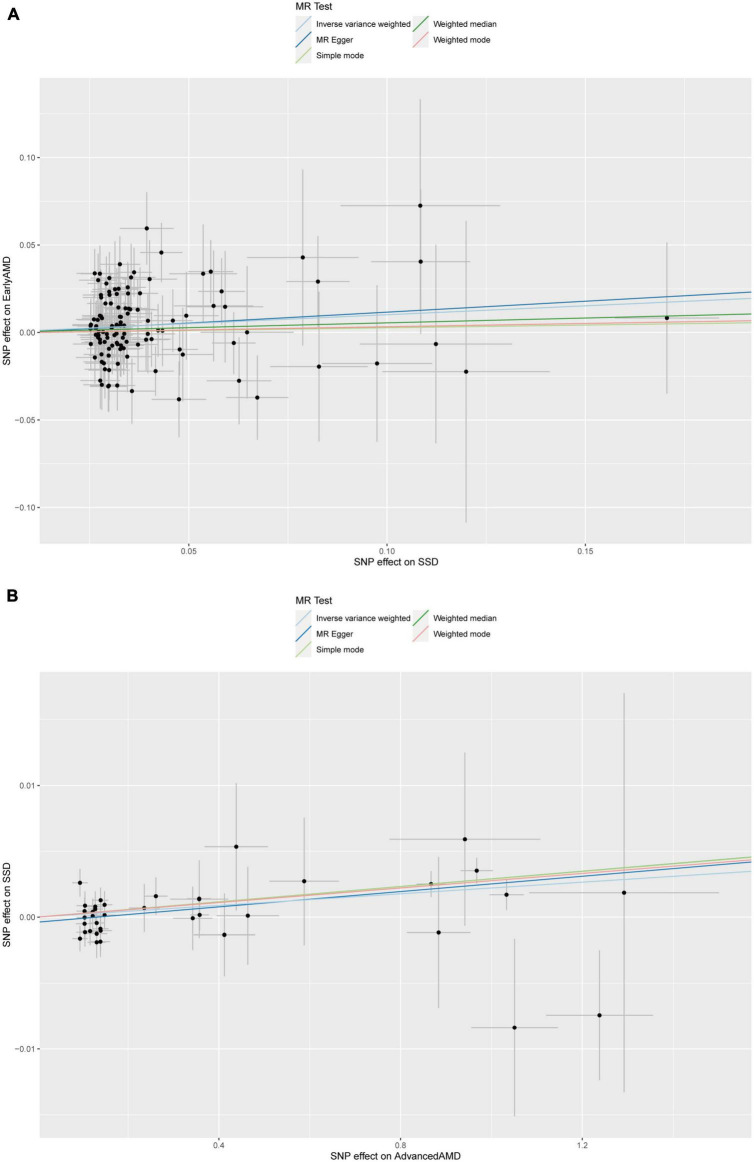
Scatter plot of **(A)** the MR estimate for the effect of SSD on the risk of early AMD; **(B)** the MR estimate for the effect of advanced AMD on the risk of SSD. SSD, short sleep duration; AMD, age-related macular degeneration; MR, Mendelian randomization; SNP, single nucleotide polymorphism.

To determine the causality and direction of the association between SD and AMD, we performed bidirectional MR analysis. For MR analysis, 8 instrumental SNPs were selected for genetically predicting early AMD (Supplementary Table 1), 23 SNPs for advanced AMD (Supplementary Table 2), 117 SNPs for SSD (Supplementary Table 3), and 27 SNPs for LSD (Supplementary Table 4). The *F* statistics for all of these genetic instruments exceeded the threshold of 10 (Pierce et al., 2011), suggesting that they were strong enough for being instruments. Results showed that SSD causally increases the risk of early AMD (β = 0.102, 95% CI: 0.008–0.195, IVW-derived *P* = 0.003) (Figure 3A and Table 5). In addition, advanced AMD causally increases the risk of SSD (β = 0.002, 95% CI: 0.001–0.003, IVW-derived *P* = 6.70E-05) (Figure 3B and Table 5). These two MR estimates all passed Bonferroni correction (IVW-derived *P* < 8.33 × 10^–3^, significant). A previous study has used MR approaches to evaluate the effect of SSD and LSD on advanced AMD (Grover and Sharma, 2022) and found no significant effects. The leave-one-out plots (Supplementary Figure 1), the funnel plots (Supplementary Figure 2), the MR-Egger intercept test, Cochran’s *Q* test, and MR-PRESSO global test (Table 5) all suggested no apparent heterogeneity or pleiotropy in these results.

**TABLE 5:** Mendelian randomization estimates for associations between abnormal SD and AMD.

Exposure	Outcome	β	95% CI	IVW-derived *P*	*P*-heterogeneity	*P*-intercept	*P*-global
SSD	Early AMD	0.102	0.008–0.195	0.003	0.29	0.876	0.81
LSD	Early AMD	0.361	−0.682 to 1.403	0.498	0.325	0.793	0.347
Early AMD	SSD	−0.003	−0.009 to 0.002	0.204	0.422	0.662	0.571
Advanced AMD	SSD	0.002	0.001–0.003	6.70E-05	0.483	0.172	0.469
Early AMD	LSD	0.002	−0.003 to 0.004	0.937	0.121	0.913	0.32
Advanced AMD	LSD	−5.80E-05	−9.15E-04 to 8.00E-04	0.895	0.381	0.958	0.422

SSD, short sleep duration; LSD, long sleep duration; AMD, age-related macular degeneration; CI, confidence interval; IVW, inverse variance-weighted; β, beta-coefficient; P-heterogeneity, P-value for heterogeneity using Cochran’s Q test; P-intercept, P-value for MR-Egger intercept; P-global, P-value for MR-PRESSO global test; PRESSO, Pleiotropy RESidual Sum and Outlier.

## 4. Discussion

Patients with NDDs such as AD often experience sleep disorders (Leng et al., 2019), while the association between SD and AMD has not been sufficiently reported. Clinical studies on this topic failed to include large populations and some of them showed apparently contradictory results. Pérez-Canales et al. (2016) reported a significant association between short SD and neovascular AMD through a case-control study, while Khurana et al. (2016) found that SD was not associated with neovascular AMD and longer sleep duration (LSD) was associated with geographic atrophy secondary to AMD. Hence, the relationship between SD and AMD has not been established and is needed to be determined and confirmed.

In this study, by using survey data from NHANES, cross-sectional analysis revealed that there was a significant association between SSD between AMD. However, observational studies are prone to reverse causation, residual confounding, and selective bias (Grimes and Schulz, 2002), which may negatively influence the estimates of relationship between SD and AMD. More importantly, observational studies can only draw an association but not causality and the direction. A RCT allows reliable and robust causal inferences to be drawn, but it is costly, time-consuming, and sometimes impractical to conduct one. MR approaches have opened up opportunities to assess and determine clinically associated characters for multiple diseases (Davey Smith and Hemani, 2014), which examines causal relationships between exposures and outcomes using genetic variants significantly associated with an exposure as instrumental variables. By using MR approaches, the biases and limitations mentioned above can be nicely overcome (Davey Smith and Hemani, 2014). In this study, we used the two-sample MR approaches to further explore and determine the relationship between SD and AMD. The results of MR analysis supported the results of cross-sectional analysis: SSD increases the risk of early AMD and advanced AMD could increase the risk of SSD.

Sleep deficiency has been reported to be associated with various diseases and the pathophysiological changes caused by it are gradually becoming a major public health and safety issue with high economic and social costs (Yang et al., 2022). Sleep deficiency has been associated with overactivation of microglia and neuroinflammation (Parhizkar et al., 2023). Apoptosis, an active cell death process modulated by genes in multicellular organisms, regulates the development of the body and maintains the stability of the internal environment. In recent years, studies have found that expression of apoptosis indices (such as Bax) are significantly elevated in sleep deficiency animals (Chen et al., 2014; Jeddi et al., 2018). Photoreceptors and RPE cells are highly energy-consuming cells in human retina, therefore, they are very sensitive to metabolic disorders. Notably, many studies have confirmed a causal relationship between sleep deficiency and energy metabolism disorders (Lim et al., 2018; Saner et al., 2018). In addition, Rodrigues et al. (2018) have found that sleep deficiency leads to changes in mitochondrial enzymes and decreases mitochondrial bioenergy efficiency in Drosophila. In the brain, sleep deficiency decreases mitochondrial membrane excitability and promotes the release of Cyt C, thus resulting in neuronal apoptosis (Xie et al., 2020). Studies have indicated that sleep deficiency may affect immune cells and the production of cytokines and complement, thereby causing immune system dysfunction and immune system-associated diseases (Ruiz et al., 2012; Irwin et al., 2016). Significant change in TNF-α level in rats has been observed after 20 and 30 days of chronic sleep deficiency (Fahmawi et al., 2023). In a study of sleep deficiency in 13 healthy young adults, van Leeuwen et al. (2009) found that five nights of sleep deficiency increases lymphocyte activation and pro-inflammatory cytokine production. In summary, potential mechanisms underlying the association between SSD and AMD include: induction of neuroinflammation, activation of cell apoptosis, energy metabolism disorder, and immune system dysfunction.

Sleep-awake cycle is controlled by the suprachiasmatic nucleus (SCN) of the midbrain. SCN receives photoperiodic information from intrinsic photosensitive retinal ganglionic cells (ipRGCs) in retina, through the retinal-hypothalamic pathway, and subsequently regulates the sleep-awake cycle of human body (Crislip et al., 2021). Decleva et al. (2022) reported the impaired pupil light reflex (PLR) and function of ipRGCs in patients with early and neovascular AMD. Similarly, Maynard et al. (2017) found the dysfunction of ipRGCs in patients with advanced AMD. These studies indicated that the increasing risk of SSD observed in patients with advanced AMD might be caused by ipRGCs dysfunction. Another potentially contributing factor to sleep deprivation in patients with AMD might be the significant impairment in brain function. The association between brain dysfunction and eye disorders has been widely reported. For instance, higher rates of depression have been reported in patients with AMD (Popescu et al., 2012). Moreover, depression has been associated with the alteration of sleep patterns (Casten et al., 2004). Cognitive impairment or brain dysfunction in patients with AMD undermines the life quality even leads to disability of patients, which may play a role in abnormal SDs observed in our study.

This study also has some limitations. Although we tried our best to exclude and correct potential confounders, there still were some additional factors pertaining to sleep (e.g., whether participants were shift workers or had young children) that failed to be taken into consideration in cross-sectional analysis. In addition, light exposure is tightly associated with SD and might be the confounder when evaluate the SD-AMD association. However, this factor was not included for analysis due to loss of data. Furthermore, findings from this study only reveal a significant association between SSD and AMD, but not the underlying mechanisms, which call for further researches.

## 5. Conclusion

In conclusion, by cross-sectional and MR analyses, the results provide evidence that there is a significant association between SSD and higher risk of AMD. The findings could be a reminder that we ophthalmologists should pay more attention to individuals with sleep disorders. Further in-depth studies are required to confirm our findings and elucidate the mechanisms underlying this association.

## Data availability statement

The original contributions presented in this study are included in the article/Supplementary material, further inquiries can be directed to the corresponding author.

## Ethics statement

Human subjects or animal subjects were not included in this study. This study used only publicly available, deidentified summary statistics from previously published works, making it exempt according to the Yan’an People’s Hospital Institutional Review Board. Our research adhered to the tenets of the Declaration of Helsinki.

## Author contributions

SL, HL, and ZL: conception and design. SL and ZL: data collection. SL: analysis and interpretation. HL: overall responsibility. All authors contributed to the article and approved the submitted version.
