# Supplementary material for: Sleep duration and age-related macular degeneration: a cross-sectional and Mendelian randomization study
Source: Front Aging Neurosci. 2023 Aug 22;15:1247413. doi: 10.3389/fnagi.2023.1247413 (PMC10477604; doi:10.3389/fnagi.2023.1247413)

Supplementary Material

Sleep Duration and Age-Related Macular Degeneration: A Cross-sectional and Mendelian Randomization Study

Shizhen Lei[1], Zhouyang Liu[2], Haihui Li[3, *]

*** Correspondence:** Haihui Li, lihaihui2018@163.com

1 Supplementary Tables

Supplemental Table 1. Instrumental SNPs from Winkler et al for representing genetically predicted early AMD

SNP	chr	effect_allele	other_allele	eaf	beta	se	pval	samplesize
rs3750847	10	T	C	0.2245	0.3838	0.0166	3.60E-98	105248
rs247617	16	A	C	0.3231	0.0922	0.0147	3.39E-10	105248
rs11569415	19	A	G	0.2141	0.1158	0.018	1.32E-10	105248
rs4658046	1	T	C	0.6098	-0.3213	0.014	8.52E-96	105248
rs4844620	1	A	G	0.2135	-0.0949	0.0173	3.79E-08	105248
rs547154	6	T	G	0.0882	-0.2178	0.0246	7.62E-19	105248
rs943080	6	T	C	0.5068	0.0797	0.0145	3.84E-08	105248
rs13278062	8	T	G	0.5207	0.0801	0.0142	1.64E-08	105248

Supplemental Table 2. Instrumental SNPs from IAMDGC Consortium for representing genetically predicted advanced AMD.

SNP	chr	effect_allele	other_allele	eaf	beta	se	pval	samplesize
rs10922109	1	C	A	0.777	-0.967584026	0.035372286	9.60E-95	33976
rs570618	1	G	T	0.42	0.867100488	0.031632721	2.00E-95	33976

rs121913059	1	C	T	0.997	3.009635179	0.299373116	8.90E-24	33976
rs148553336	1	T	C	0.997	-1.237874356	0.117888122	8.60E-26	33976
rs187328863	1	C	T	0.946	0.819779831	0.046804325	1.10E-68	33976
rs61818925	1	G	T	0.716	-0.510825624	0.018662716	6.00E-95	33976
rs35292876	1	C	T	0.979	0.88376754	0.069728637	8.20E-37	33976
rs191281603	1	C	G	0.993	-0.94160854	0.165969331	0.000000014	33976
rs11884770	2	C	T	0.742	-0.105360516	0.01899288	0.000000029	33976
rs62247658	3	T	C	0.534	0.131028262	0.017096224	1.80E-14	33976
rs140647181	3	T	C	0.977	0.463734016	0.06862096	1.40E-11	33976
rs55975637	3	G	A	0.868	0.139761942	0.02457973	0.000000013	33976
rs10033900	4	C	T	0.489	0.139761942	0.016682736	5.40E-17	33976
rs141853578	4	C	T	0.997	1.291983682	0.208966333	6.30E-10	33976
rs62358361	5	G	T	0.984	0.587786665	0.07627822	1.30E-14	33976
rs114092250	5	G	A	0.984	-0.356674944	0.063651552	0.000000021	33976
rs116503776	6	G	A	0.91	-0.562118918	0.026001604	1.20E-93	33976
rs144629244	6	G	A	0.984	1.026041596	0.086120306	1.00E-32	33976
rs114254831	6	A	G	0.716	0.122217633	0.017932562	9.40E-12	33976
rs181705462	6	G	T	0.982	0.438254931	0.069633983	3.10E-10	33976
rs943080	6	T	C	0.535	-0.127833372	0.016543452	1.10E-14	33976
rs1142	7	C	T	0.63	0.104360015	0.01723398	1.40E-09	33976
rs7803454	7	C	T	0.791	0.122217633	0.020877765	4.80E-09	33976
rs79037040	8	T	G	0.549	-0.105360516	0.015996182	4.50E-11	33976
rs10781182	9	G	T	0.672	0.104360015	0.017524639	2.60E-09	33976
rs71507014	9	GC	G	0.573	0.09531018	0.017199543	0.00000003	33976

rs1626340	9	G	A	0.811	-0.127833372	0.020414049	3.80E-10	33976
rs2740488	9	A	C	0.745	-0.105360516	0.018485189	0.000000012	33976
rs12357257	10	G	A	0.757	0.104360015	0.019064687	0.000000044	33976
rs3750846	10	T	C	0.564	1.033184483	0.037750821	6.50E-95	33976
rs3138141	12	C	A	0.778	0.148420005	0.025274943	4.30E-09	33976
rs61941274	12	G	A	0.976	0.412109651	0.067623481	1.10E-09	33976
rs9564692	13	C	T	0.723	-0.116533816	0.018544569	3.30E-10	33976
rs61985136	14	T	C	0.64	-0.105360516	0.016474108	1.60E-10	33976
rs2842339	14	A	G	0.893	0.131028262	0.023188871	0.000000016	33976
rs2043085	15	T	C	0.65	-0.139262067	0.017749754	4.30E-15	33976
rs2070895	15	G	A	0.805	-0.139262067	0.020849635	2.40E-11	33976
rs5817082	16	C	CA	0.768	-0.174353387	0.01948386	3.60E-19	33976
rs17231506	16	C	T	0.652	0.148420005	0.016969002	2.20E-18	33976
rs72802342	16	C	A	0.933	-0.235722334	0.03413507	5.00E-12	33976
rs11080055	17	C	A	0.537	-0.094310679	0.016457013	0.00000001	33976
rs6565597	17	C	T	0.6	0.122217633	0.01811195	1.50E-11	33976

Supplemental Table 3. Instrumental SNPs for representing genetically predicted short sleep duration (SSD).

**SNP**	**chr**	**effect_allele**	**other_allele**	**eaf**	**beta**	**se**	**pval**	**samplesize**
rs10067113	5	C	T	0.380528	-0.0280609	0.00465694	2.50E-09	446118
rs10123584	9	G	A	0.292722	0.0270426	0.00495784	4.00E-08	446118

rs10149448	14	A	G	0.603659	0.0272319	0.00461608	5.60E-09	446118
rs10196909	2	C	A	0.481825	0.0321137	0.00450856	6.20E-13	446118
rs1027742	5	A	G	0.738161	0.0306919	0.00514017	3.60E-09	446118
rs10495976	2	A	T	0.609803	-0.0280661	0.00464114	8.00E-10	446118
rs10501087	11	T	C	0.799452	-0.0346935	0.00562434	5.40E-10	446118
rs10520176	2	T	C	0.501844	0.0333663	0.00451125	2.00E-13	446118
rs10818834	9	T	C	0.733086	0.0323011	0.00511442	4.20E-10	446118
rs10976942	9	C	A	0.917117	-0.0493619	0.00817039	1.00E-09	446118
rs11032362	11	G	A	0.909159	-0.0672744	0.0078366	7.30E-18	446118
rs11152350	18	A	C	0.470982	-0.0279869	0.00451706	6.40E-10	446118
rs11174781	12	T	C	0.877633	0.0475212	0.00687518	3.90E-12	446118
rs11229543	11	G	A	0.759944	0.033727	0.00527519	1.10E-10	446118
rs1144566	1	T	C	0.0301951	0.170568	0.0131392	2.80E-38	446118
rs114870822	2	G	A	0.987205	-0.108373	0.0201178	3.40E-08	446118
rs115774037	3	T	C	0.973872	-0.078752	0.0140393	3.50E-08	446118
rs11580135	1	T	C	0.317102	-0.0275921	0.00483431	1.30E-08	446118
rs11587758	1	G	A	0.604254	-0.0346078	0.00459127	3.80E-14	446118
rs11588913	1	G	A	0.601488	0.0252376	0.00459169	2.50E-08	446118
rs11645898	16	T	C	0.832897	0.0356701	0.00604233	1.80E-09	446118
rs11670534	19	C	T	0.834403	0.0328734	0.00608396	4.70E-08	446118
rs11679484	2	C	A	0.626068	-0.0298469	0.00467197	1.60E-10	446118
rs11712056	3	T	C	0.556466	0.031956	0.0045329	3.70E-12	446118
rs11786306	8	G	C	0.646069	-0.0292175	0.00474691	7.00E-10	446118
rs11841335	13	G	A	0.746028	0.0283759	0.00518535	4.00E-08	446118
rs12055234	5	G	A	0.67152	-0.0271463	0.00480045	1.50E-08	446118
rs12140153	1	G	T	0.904974	0.0484879	0.00787657	4.80E-10	446118
rs12669911	7	A	C	0.384427	-0.0294686	0.00466454	1.80E-10	446118

rs12682033	8	T	C	0.240285	0.034887	0.00529299	6.10E-11	446118
rs12927162	16	A	G	0.721652	0.0459823	0.00503141	2.00E-20	446118
rs12969848	18	C	T	0.470293	-0.0362004	0.00452995	2.60E-15	446118
rs13059636	3	A	G	0.528035	-0.0317863	0.0045329	3.50E-12	446118
rs13065394	3	G	T	0.711759	0.0297983	0.00497014	2.20E-09	446118
rs13255030	8	A	G	0.58516	0.026079	0.00457953	1.50E-08	446118
rs13269289	8	G	A	0.673433	-0.0275791	0.00485097	5.50E-09	446118
rs138964083	2	C	T	0.941426	-0.0591602	0.00960026	1.00E-09	446118
rs139911	22	C	T	0.42411	0.0377065	0.00457846	2.20E-16	446118
rs1421085	16	T	C	0.59687	-0.0416273	0.00459528	1.80E-19	446118
rs1470764	8	G	A	0.387169	0.0285814	0.00463098	5.90E-10	446118
rs1494185	7	G	A	0.725563	0.028783	0.00505302	1.10E-08	446118
rs149611468	3	T	C	0.98802	0.119935	0.0211151	1.30E-08	446118
rs1524472	7	A	G	0.440956	0.0252952	0.00453478	2.10E-08	446118
rs17374439	1	C	T	0.803508	-0.0555478	0.00566145	1.20E-22	446118
rs17575798	1	G	A	0.806886	0.0392352	0.00568886	7.80E-12	446118
rs17682747	17	G	A	0.767481	-0.0305453	0.00534573	8.70E-09	446118
rs1947198	2	C	T	0.87783	-0.0396714	0.00686993	3.20E-09	446118
rs202157	7	C	T	0.29887	0.0397113	0.00493645	1.30E-15	446118
rs2072727	20	T	C	0.4361	0.0278367	0.0045507	5.60E-10	446118
rs2102506	3	G	A	0.3605	0.0293301	0.00472786	8.30E-10	446118
rs2239626	3	T	C	0.695468	-0.0335827	0.00490904	5.00E-12	446118
rs231398	4	G	A	0.837855	0.0354509	0.00615616	5.80E-09	446118
rs2467109	12	T	A	0.718089	-0.0276799	0.00501668	3.90E-08	446118
rs2518022	17	T	C	0.0835266	0.0626693	0.00813849	7.60E-15	446118
rs2653343	6	T	A	0.214721	0.0613434	0.00549376	5.20E-29	446118
rs28380327	2	A	T	0.629168	0.0289857	0.0046586	7.80E-10	446118
rs2842638	6	T	G	0.576697	0.0306548	0.0046083	1.90E-11	446118

rs28634184	4	C	T	0.749415	0.0299077	0.00521122	1.30E-08	446118
rs2893787	10	G	A	0.2553	0.0288453	0.00516506	3.50E-08	446118
rs2910032	5	C	T	0.482294	-0.0336963	0.00451163	1.10E-13	446118
rs2949923	17	A	G	0.538655	-0.0265539	0.0045291	4.20E-09	446118
rs308521	9	T	C	0.602903	0.0279091	0.00461881	1.50E-09	446118
rs34581681	3	G	A	0.839317	0.034486	0.00617986	4.20E-08	446118
rs34619169	9	G	A	0.692101	-0.0272806	0.00488632	1.50E-08	446118
rs34627176	4	G	A	0.786135	-0.0298813	0.00551285	3.60E-08	446118
rs34875688	5	T	A	0.767268	0.0320299	0.00534073	1.10E-09	446118
rs35653190	17	C	T	0.771778	0.0303562	0.00540798	2.00E-08	446118
rs3760185	17	C	T	0.751597	0.0371222	0.00527711	2.10E-12	446118
rs3767240	1	T	C	0.6179	-0.0312547	0.00462739	7.30E-12	446118
rs3850174	3	T	A	0.742864	0.0318495	0.00519636	1.40E-09	446118
rs3877930	6	A	G	0.986059	-0.112315	0.0192049	3.00E-09	446118
rs4239386	18	T	A	0.665044	0.0343168	0.00478623	4.20E-13	446118
rs4241964	4	T	G	0.525037	-0.0326407	0.00453345	7.20E-13	446118
rs4321976	8	T	C	0.778752	0.0328285	0.00543138	8.70E-10	446118
rs4339281	4	A	G	0.872986	0.0394057	0.00677257	5.30E-09	446118
rs4752593	10	G	C	0.371575	0.0265676	0.00468542	9.80E-09	446118
rs4822107	22	G	A	0.494268	-0.0278435	0.00453576	8.70E-10	446118
rs4936291	11	A	G	0.610604	-0.0276435	0.00474862	9.40E-09	446118
rs520954	6	A	G	0.672827	-0.0422581	0.00479665	1.60E-18	446118
rs60194061	3	G	A	0.73032	-0.0326372	0.00511118	8.60E-11	446118
rs60616179	6	A	G	0.945063	0.0562524	0.009965	9.20E-09	446118
rs62046253	16	C	T	0.655839	-0.0299674	0.00473879	1.40E-10	446118
rs62082401	18	C	G	0.809399	-0.0433691	0.00574168	1.20E-14	446118
rs62553781	9	C	T	0.965073	0.0828273	0.0123021	3.00E-11	446118
rs6537834	1	T	C	0.385278	-0.0252152	0.00462393	3.50E-08	446118
rs6599694	10	G	T	0.656185	0.0266711	0.00476984	2.10E-08	446118

rs6656331	1	C	T		0.475922	0.0271244	0.00451193	1.50E-09	446118
rs6744983	2	G	T		0.62256	-0.0253244	0.00464336	4.90E-08	446118
rs6967481	7	C	T		0.50325	-0.029923	0.00452547	1.90E-11	446118
rs7001604	8	T	C		0.613053	-0.0318374	0.00465169	7.30E-12	446118
rs72632979	11	A	G		0.828665	0.0346051	0.00599706	4.00E-09	446118
rs72720396	1	A	G		0.769471	-0.0430894	0.00533877	5.30E-16	446118
rs72829936	17	G	A		0.836049	-0.0352298	0.00610979	5.50E-09	446118
rs7302062	12	T	C		0.55303	0.0317771	0.00453388	5.20E-12	446118
rs7304278	12	A	G		0.274962	-0.0327762	0.00506843	9.60E-11	446118
rs7313852	12	G	A		0.564644	0.0477061	0.00455758	3.80E-25	446118
rs73606718	11	G	A		0.879064	0.040119	0.00691498	5.70E-09	446118
rs75120545	2	C	T		0.969822	-0.0974698	0.013897	2.90E-12	446118
rs7547493	1	A	G		0.82194	-0.0582789	0.00587503	2.50E-23	446118
rs75650221	1	C	T		0.961665	-0.0646983	0.011744	3.30E-08	446118
rs7602425	2	C	T		0.924493	-0.0536212	0.00854512	3.20E-10	446118
rs769066	8	T	C		0.816231	-0.0329101	0.00583263	1.90E-08	446118
rs7691121	4	C	G		0.767925	0.0340511	0.00532954	1.00E-10	446118
rs77008212	2	A	G		0.912884	0.0825698	0.00798588	1.10E-24	446118
rs7701529	5	A	T		0.237701	-0.0300077	0.00532109	1.70E-08	446118
rs7735794	5	G	A		0.775914	-0.0313895	0.00570769	4.50E-08	446118
rs778147	2	C	A		0.366787	0.0296842	0.00466945	4.60E-10	446118
rs78095690	20	T	C		0.564251	-0.0275925	0.00456112	1.10E-09	446118
rs786406	2	A	G		0.297822	-0.0327319	0.00492279	1.00E-11	446118
rs7959983	12	T	C		0.595837	-0.0318282	0.00457585	4.70E-12	446118
rs9365769	6	A	G		0.436791	0.0262836	0.00457414	7.30E-09	446118
rs9369915	6	G	A		0.694286	-0.0371942	0.00491617	3.00E-14	446118
rs9573971	13	A	G		0.966149	0.108483	0.0124737	4.90E-18	446118
rs957501	3	T	A		0.336033	0.0263167	0.00477453	3.00E-08	446118

rs9597241	13	A	C	0.811186	0.0322479	0.00579356	2.70E-08	446118

Supplemental Table 4. Instrumental SNPs for representing genetically predicted long sleep duration (LSD).

**SNP**	**chr**	**effect_allele**	**other_allele**	**eaf**	**beta**	**se**	**pval**	**samplesize**
rs61773390	1	G	T	0.803561	-0.0340792	0.00337553	1.20E-23	446118
rs17448682	1	C	T	0.768048	-0.0221528	0.00318247	2.20E-12	446118
rs3816454	1	T	G	0.374768	0.020082	0.00278156	4.90E-13	446118
rs12140153	1	G	T	0.904737	0.0340109	0.00468803	1.60E-13	446118
rs7547493	1	A	G	0.821984	-0.0376299	0.00350319	3.90E-27	446118
rs6658041	1	G	A	0.40008	-0.0152482	0.00274124	1.70E-08	446118
rs11588913	1	G	A	0.601681	0.0154525	0.00273856	1.50E-08	446118
rs72720396	1	A	G	0.769656	-0.0271261	0.00318521	8.40E-18	446118
rs4949980	1	A	G	0.932393	-0.0321893	0.00539402	2.70E-09	446118
rs17575798	1	G	A	0.807172	0.0225553	0.00339377	4.20E-11	446118
rs11587758	1	G	A	0.604485	-0.0241103	0.0027375	5.50E-19	446118
rs140206235	1	C	T	0.980731	-0.0553854	0.00996801	2.30E-08	446118
rs975025	1	C	T	0.922916	0.0321062	0.00502525	1.30E-10	446118
rs509476	1	T	C	0.0301569	0.1137	0.0078575	5.70E-47	446118
rs13011556	2	C	G	0.761404	-0.0227424	0.00316075	1.10E-12	446118
rs2712056	2	C	T	0.815336	-0.022175	0.00345992	5.10E-11	446118
rs75120545	2	C	T	0.969821	-0.0613917	0.00828587	1.10E-13	446118
rs786406	2	A	G	0.297873	-0.0235784	0.00293536	1.90E-16	446118
rs10495976	2	A	T	0.609779	-0.0178512	0.00276698	5.10E-11	446118
rs7602425	2	C	T	0.924418	-0.031168	0.00509212	8.50E-10	446118
rs359250	2	G	T	0.363664	-0.0168642	0.0027885	9.20E-10	446118
rs812925	2	C	G	0.648756	-0.0205641	0.00280786	6.90E-13	446118

rs2311837	2	T	C	0.591721	-0.0151285	0.00272441	3.60E-08	446118
rs2422413	2	T	C	0.622358	-0.0162894	0.0027689	3.60E-09	446118
rs10520176	2	T	C	0.50187	0.0231498	0.00268932	1.40E-17	446118
rs13255030	8	A	G	0.585145	0.0162198	0.00273214	2.80E-09	446118
rs12541362	8	A	T	0.649758	-0.0213568	0.00282446	3.50E-14	446118

2 Supplementary Figures

Supplementary Figure 1. Leave-one-out plots of estimates for the effect of (A) SSD on the risk of early AMD; (B) advanced AMD on the risk of SSD. SSD: short sleep duration; AMD: age-related macular degeneration; MR: Mendelian randomization.

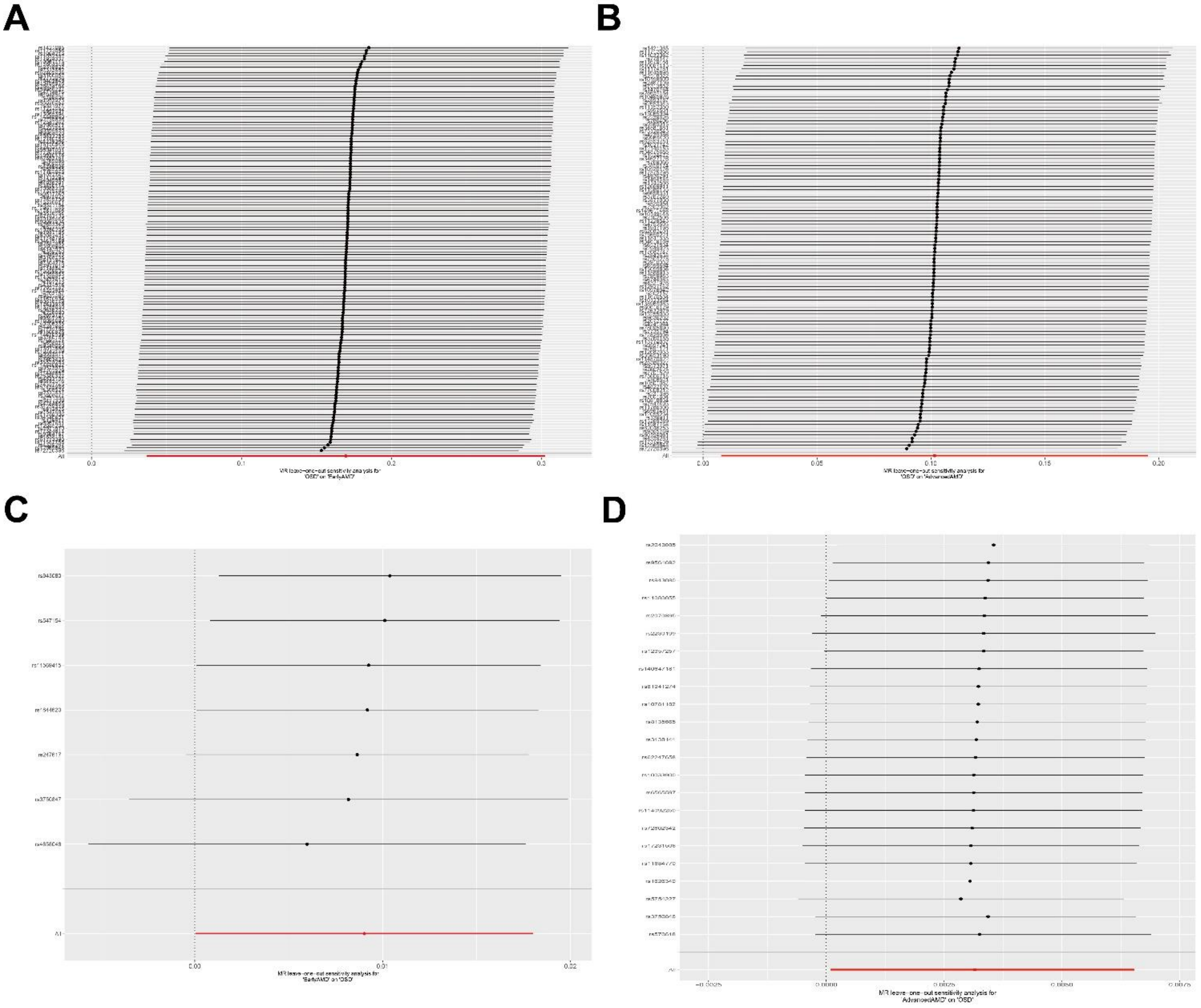

Supplementary Figure 2. Funnel plots of estimates for the effect of (A) SSD on the risk of early AMD; (B) advanced AMD on the risk of SSD. SSD: short sleep duration; AMD: age-related macular degeneration; MR: Mendelian randomization.

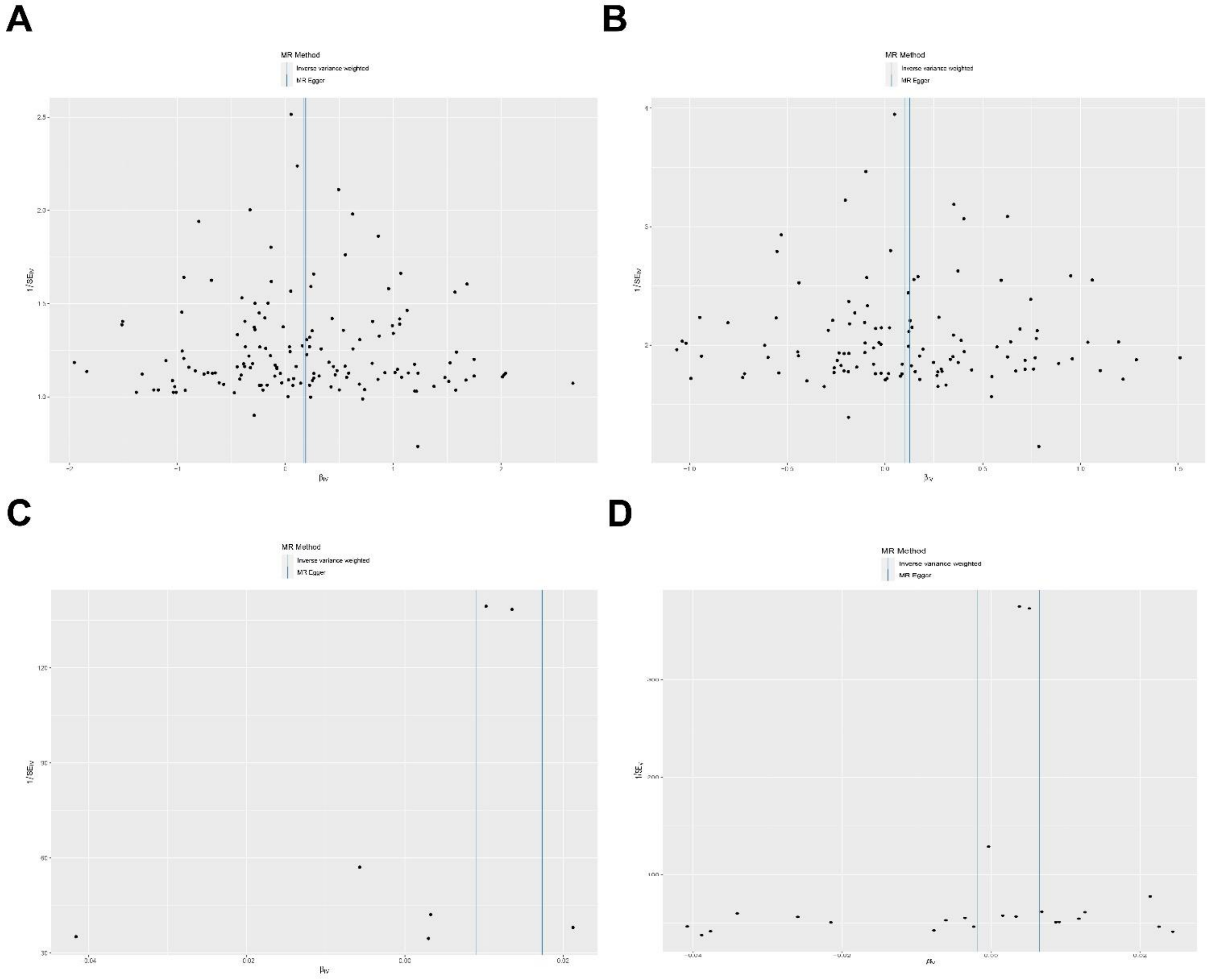

Supplement: Supplementary file 1 [file Data_Sheet_1.PDF]
